# Prenatal Choline Supplementation Improves Glucose Tolerance and Reduces Liver Fat Accumulation in Mouse Offspring Exposed to Ethanol during the Prenatal and Postnatal Periods

**DOI:** 10.3390/nu16091264

**Published:** 2024-04-24

**Authors:** Isma’il Kadam, Steven E. Trasino, Hunter Korsmo, Jessica Lucas, Myriam Pinkas, Xinyin Jiang

**Affiliations:** 1 PhD Program in Biochemistry, Graduate Center of the City University of New York, New York, NY 10016, USA; ikadam@gradcenter.cuny.edu (I.K.); hkorsmo@gradcenter.cuny.edu (H.K.); 2 Department of Health and Nutrition Sciences, Brooklyn College of the City University of New York, Brooklyn, NY 11210, USA; jessica.lucas00@bcmail.cuny.edu (J.L.); myriampinkas@gmail.com (M.P.); 3 Nutrition Program, School of Urban Public Health, Hunter College, City University of New York, New York, NY 10065, USA

**Keywords:** prenatal alcohol exposure, choline, steatosis, glucose tolerance

## Abstract

Prenatal alcohol exposure (AE) affects cognitive development. However, it is unclear whether prenatal AE influences the metabolic health of offspring and whether postnatal AE exacerbates metabolic deterioration resulting from prenatal AE. Choline is a semi-essential nutrient that has been demonstrated to mitigate the cognitive impairment of prenatal AE. This study investigated how maternal choline supplementation (CS) may modify the metabolic health of offspring with prenatal and postnatal AE (AE/AE). C57BL/6J female mice were fed either a Lieber–DeCarli diet with 1.4% ethanol between embryonic day (E) 9.5 and E17.5 or a control diet. Choline was supplemented with 4 × concentrations versus the control throughout pregnancy. At postnatal week 7, offspring mice were exposed to 1.4% ethanol for females and 3.9% ethanol for males for 4 weeks. AE/AE increased hepatic triglyceride accumulation in male offspring only, which was normalized by prenatal CS. Prenatal CS also improved glucose tolerance compared to AE/AE animals. AE/AE suppressed hepatic gene expression of peroxisome proliferator activated receptor alpha (*Ppara*) and low-density lipoprotein receptor (*Ldlr*), which regulate fatty acid catabolism and cholesterol reuptake, respectively, in male offspring. However, these changes were not rectified by prenatal CS. In conclusion, AE/AE led to an increased risk of steatosis and was partially prevented by prenatal CS in male mice.

## 1. Introduction

Although alcohol is a well-known cause of fetal alcohol spectrum disorders (FASDs), about 15–75% of pregnant women in different populations admit to consuming alcoholic drinks and 4–30% of them have risky drinking episodes (drinking three or more units of alcohol in a single occasion) during pregnancy [1,2,3,4]. At birth, FASD is characterized by low birth weight or growth deficiency, craniofacial and neural abnormalities, while in the long term, it is associated with learning and memory impairment and increased incidence of mental illnesses in both animal and human studies [1,2,3,4,5,6].

The Developmental Origins of Health and Disease (DOHaD) theory indicates that a growth-restricted environment during the prenatal period increases the risk of cardiometabolic diseases later in life [7]. Evidence regarding PAE’s lasting impacts on metabolic health has begun to emerge. One study has demonstrated that providing pregnant guinea pigs with 4 g/kg alcohol per day throughout gestation results in growth restriction at birth but rapid postnatal catch-up growth and increased adiposity after birth, and the excess adiposity was exacerbated by a postnatal obesogenic environment due to high-fat (HF) feeding [8]. Giving rats the same dose of alcohol during gestational day 11–20 leads to higher cholesterol levels in the offspring [9]. PAE also results in glucose intolerance in rat offspring and this phenotype appears to be transmitted intergenerationally [10,11,12].

Alarmingly, animal studies suggest that PAE increases young offspring’s acceptance of ethanol [13,14], while several epidemiological studies suggest that PAE is associated with early drinking behavior and increased risk of alcohol abuse in adolescence and adulthood [15,16,17]. It is largely unexplored whether chronic exposure to ethanol both prenatally and postnatally may exacerbate cardiometabolic dysfunctioning and the risk of developing alcoholic liver disease (ALD) later in life.

As the main site of oxidative alcohol metabolism, the influence of ethanol on the liver’s lipid metabolism is especially prominent through replacing fat as an energy source, thereby sparing lipids for accumulation, increasing the expression of lipogenic genes, and reducing reverse transport of cholesterol-containing lipoproteins [9,18]. Ethanol increases the exhaustion of nutrients involved in one-carbon metabolism, such as folate, vitamin B_12_ and choline, which are important for biosynthesis including lipoproteins that mediate export of triglycerides from the liver, as well as epigenetic modifications of lipid metabolic genes [19]. Ethanol deprives glutathione due to heightened oxidative stress, thereby reducing the breakdown of homocysteine through transsulfuration which requires glutathione. This results in the decrease in S-adenosylmethionine (SAM) needed for methyl group supply for DNA methylation [20]. Consistently, DNA hypomethylation was seen in alcohol-fed mouse livers, which was rectified by methyl donor supplementation [21,22,23].

Choline is both a liver lipotrope and a methyl donor. In animals, prenatal AE-related neurocognitive problems such as spatial memory and learning deficits were observed to be alleviated by choline supplementation (CS) during pregnancy [24,25,26,27]. These findings corroborate a handful of randomized controlled trials (RCTs) among heavy drinking pregnant women, which demonstrate that prenatal CS improved recognition memory of affected children [28,29]. Previous studies of our group demonstrated the benefit of prenatal CS on lipid metabolism and cardiometabolic health, including alleviation of excess adiposity and liver fat accumulation in fetuses and improving glucose tolerance after postnatal exposure to HF feeding [30,31].

The current study aimed to investigate whether exposure to ethanol both prenatally and postnatally exacerbates metabolic disturbance and whether prenatal CS can alleviate the negative impact of AE.

## 2. Materials and Methods

### 2.1. Animals and Diets

**Figure 1:**
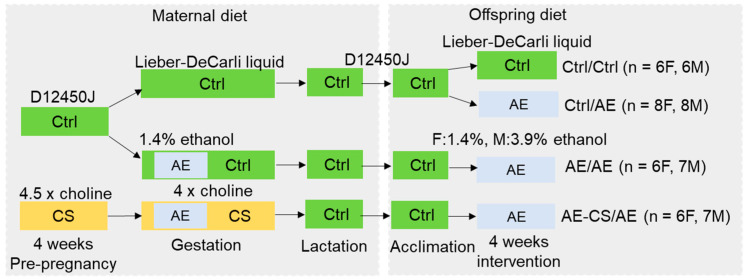
Study design. C57BL/6J female mice received either a control or choline-supplemented diet with or without ethanol during pregnancy. Their offspring received either a control or ethanol-containing Lieber–DeCarli liquid diet after weaning for 4 weeks. AE: alcohol exposure; CS: choline supplementation; Ctrl: control. F: female, M: males; *n* = 6–8 offspring/sex/treatment group from 5 dams per group.

The study protocol was approved by the Institutional Animal Care and Use Committee (IACUC) at Brooklyn College. C57BL/6J mice were obtained from the Jackson Laboratory (Farmington, CT, USA) originally and were bred in the Brooklyn College animal facility. The mice were housed at 22 °C, humidity 40–60%, and a 12 h light/dark cycle with regular bedding and enrichment. At 6 weeks of age, the female mice were fed a semi-chemically defined control mouse diet (D12450J, Research Diets, New Brunswick, NJ, USA) ad libitum while receiving either 25 mM choline chloride or plain drinking water for 4 weeks (Figure 1). We previously found that this amount of supplementation provided about 4.5 times choline intake in the supplemented versus control group [32]. Male mice also received the D12450J diet and plain drinking water. Dietary composition was described previously [32].

After 4 weeks of feeding with experimental diets, female and male mice were caged together in a 2:1 ratio for timed mating. If a vaginal plug was detected in the morning, the female mouse was transferred to a separate cage and time was recorded as embryonic day (E) 0.5. They received a Lieber–DeCarli’82 control liquid diet (Bio-serv, Flemington, NJ, USA) with or without 830 mg/L choline chloride (providing 619 mg/L choline) until E8.5. The supplemental amount of choline chloride plus the original choline contained in the liquid diet (206 mg/L) provided 4 times the choline intake compared to the unsupplemented group. During E9.5 to E17.5 of gestation, a subset of animals received the Lieber–DeCarli’82 Ethanol liquid diet (Bio-serv) with 1.4% (*w*/*v*%) pure ethanol and the others continued on the original liquid diet without ethanol. This level of ethanol exposure provided approximately 10% of total calories derived from ethanol, which was equivalent to about 2.5–3 drinks per day that exceeded the hepatotoxicity threshold of 2 drinks per day for women. Thereafter, the dams were switched back to D12450J until weaning of pups. After weaning (postnatal day PD21), pups were given the D12450J control diet for 2 weeks, then acclimated to a Lieber–DeCarli control liquid diet without alcohol for 2 weeks. Thereafter, part of the male pups received an alcohol-containing Lieber–DeCarli diet (3.9% *w*/*v*) while the female pups received an alcohol-containing Lieber–DeCarlil diet (1.4% *w*/*v*) for 4 weeks, a duration that was considered sufficient to trigger early signs of alcoholic liver disease such as steatosis and liver enzyme elevation [33]. The difference in concentrations of ethanol was due to the different levels of tolerance to ethanol for male and female animals determined by a trial run, which exposed non-pregnant mice to 1.4%, 2%, 3%, 3.9%, and 5% ethanol. Animals experienced drastic weight loss, deteriorated activity, and abnormal behavior after AE of concentrations higher than 3.9% and 1.4% in males and females, respectively. The 3.9% ethanol diet provided male offspring with about 28% of total calories from ethanol, approximating 7–8 drinks per day that exceeded the 4-drink per day hepatotoxicity threshold for men. The Lieber–DeCarli’82 control diet was designed to be isocaloric to the Lieber–DeCarli’82 ethanol diet containing 5% ethanol. Since we added only 1.4% and 3.9% ethanol to the diet, we used maltose dextrin to increase the caloric density of AE groups in place of ethanol to make the diets isocaloric to the control group according to the manufacturer’s instructions. Supplementary Table S1 describes the composition of the liquid diets used. We prepared fresh liquid diets each day due to the instability and increased risk of contamination of these diets over time. Liquid food intake was assessed with the decrease in fluid volume each day and weight of offspring was measured using an analytical balance every week during the 4 weeks of postnatal liquid diet feeding.

After alcohol feeding for 4 weeks, offspring mice were fasted for 6 h starting at 9 a.m. and an intraperitoneal glucose tolerance test (GTT) was conducted as previously described [32]. On the next day, mice were euthanized by carbon dioxide inhalation after 6 h fasting starting from 9 a.m. Immediately after euthanasia, cardiac puncture was conducted to retrieve blood. Blood samples were allowed to clot in a serum separator tube (BD, Franklin Lakes, NJ, USA). The blood was centrifuged at 12,000× *g* for 10 min to obtain serum. Livers and gonadal fat surrounding reproductive organs were dissected, rinsed in phosphate-buffered saline, and dried on absorbent paper. The samples were then weighed on an analytical balance. Thereafter, they were fixed in 10% formalin, flash-frozen in liquid nitrogen and stored at −80 °C, or immersed in RNAlater^®®^ (Thermo Scientific, Grand Island, NY, USA) overnight before being stored at −80 °C until analysis. We included 1–2 pups/sex in each litter for each dietary treatment to reach 6–8 pups per sex per treatment group for analyses.

### 2.2. RNA Extraction and Quantitative Real-Time PCR

RNA was extracted from fetal livers using the TRIzol^®®^ reagent (Thermo Fisher Scientific, Waltham, MA, USA) with DNAase I (Qiagen, Germantown, MA, USA) treatment to remove genomic DNA. Reverse transcription was conducted using the High-Capacity cDNA Reverse Transcription kit (Thermo Fisher Scientific, Waltham, MA, USA) following the manufacturer’s instructions. Gene transcript abundance was analyzed by quantitative real-time PCR with SYBR green detection using the CFX384 Touch™ Real-Time PCR Detection System (Bio-Rad, Hercules, CA, USA) as previously reported. Data for each gene of interest was normalized as the fold difference to the housekeeping gene beta-actin (*Actb*) using the ΔΔCt method and then expressed as the fold difference to the control group without ethanol exposure at any point of life (Ctrl/Ctrl) [31]. Primers were designed using GeneRunner Version 3.01 (http://www.softpedia.com, accessed on 1 December 2023) as previously published [30,31,32]. The primers expanded over two exons to avoid amplification of genomic DNA. Amplification specificity was assessed by the single peak in the melt curve. Each gene for each sample was run in triplicates. Expression of the following genes was analyzed: fatty acid synthase (*Fasn*) that mediates fatty acid de novo synthesis; peroxisome proliferator-activated receptor alpha (*Ppara*), carnitine palmitoyltransferase I (*Cpt1a*) and acyl-CoA oxidase 1 (*Acox1*) that mediates fatty acid β oxidation; cluster of differentiation 36 (*Cd36*) that mediates fatty acid transport; lipoprotein lipase (*Lpl*) that mediates fatty acid extraction from lipoprotein, and low-density lipoprotein receptor (*Ldlr*) that mediates lipoprotein uptake. We also measured the expression of genes involved with choline metabolism, including choline-phosphate cytidylyltransferase (*Pcyt1a*), phosphatidylethanolamine *N*-methyltransferase (*Pemt*), and betaine-homocysteine *S*-methyltransferase 1 (*Bhmt1*).

### 2.3. Liver Triglyceride (TG) and Malondialdehyde (MDA) Measurements

Liver tissues were homogenized and analyzed with the triglyceride colorimetric assay kit according to the manufacturer’s instructions (Cayman, Ann Arbor, MI, USA) and the TG concentrations were normalized with the weight of the tissue sample. Lipid peroxide levels were measured from MDA using the TBARs TCA Method Assay Kit (Cayman Chemical, Ann Arbor, MI, USA).

### 2.4. Serum Measurements

Serum biomarkers were measured with assay kits according to the manufacturers’ instructions. TG was measured with the triglyceride colorimetric assay kit (Cayman, Ann Arbor, MI, USA); free fatty acids (FFAs) were measured with the HR Series NEFA-HR(2) colorimetric reagents (Wako Diagnostics, Richmond, VA, USA); apolipoprotein B (ApoB) levels were analyzed with the ApoB ELISA kit (MyBioSource, San Diego, CA, USA); serum ALT levels were measured with the ALT (SGPT) reagent set (Teco Diagnostics, Anaheim, CA, USA).

### 2.5. Choline Measurements

Whole fetal livers were used for choline extraction and quantification. Measurements of choline and its derivatives were conducted using LC-MS/MS methodology [34].

### 2.6. Histology

Liver samples fixed with 10% formalin were sent to HistoWiz Inc. (Brooklyn, NY, USA) for further processing. The samples were embedded in paraffin, cut in 5 µm sections, and stained with H&E. Slides were then scanned from 1× to 200× magnification and converted to analyzable electronic files. The liver slides were examined for signs of steatosis and inflammation and scored with the nonalcoholic fatty liver disease activity scores (NAS) by Brunt et al. [35]. Briefly, H&E-stained liver sections were evaluated with the following histological features: ductular reaction (score 0–3), portal and lobular inflammation (0: none, 1: mild, 2: moderate; 3: severe), presence or absence of atypical cells comprising the ductular reaction, degree of steatosis (0: none, 1 <30%, 2 >30 but <60%, 3- >60%), type of steatotic vacuoles (microvesicular or macrovesicular) and ballooning degeneration (0: none, 1: occasional, 2: more than occasional, 3: numerous cells).

### 2.7. Global DNA Methylation

DNA was extracted from samples using the GeneJET Genomic DNA Purification Kit (Thermo Fisher, Suwanee, GA, USA) following the manufacturer’s instructions. DNA quantity and quality was determined by a Nanodrop machine (Thermo Fisher). An A260/280 ratio over 1.8 was considered acceptable. Thereafter, the isolated DNA was treated with nuclease P1, alkaline phosphatase, and phosphodiesterase (Sigma-Aldrich, St. Louis, MO, USA) [36]. CpG methylation of the samples was quantified with a DNA methylation ELISA kit (Cayman Chemical, Ann Arbor, MI, USA) following the manufacturer’s instructions.

### 2.8. Statistical Analysis

Analysis of Variance (ANOVA) tests followed by post hoc least significant difference (LSD) tests were constructed to assess the differences in the dependent variables among the dietary treatment groups. Given the sexually dimorphic response in energy metabolism and different dosages of ethanol exposure, we analyzed the data in the two sexes separately. Dependent variables with residuals that deviate from the normal distribution were logarithmically transformed before analysis. A *p* value < 0.05 was considered significant. Values are presented as means ± standard error of the mean (SEM). Data were analyzed with the SPSS software (version 24, IBM Inc., Armonk, NY, USA).

## 3. Results

### 3.1. Weight Gain and Adiposity Were Lower in Male Offspring Exposed to Ethanol after Weaning

**Figure 2:**
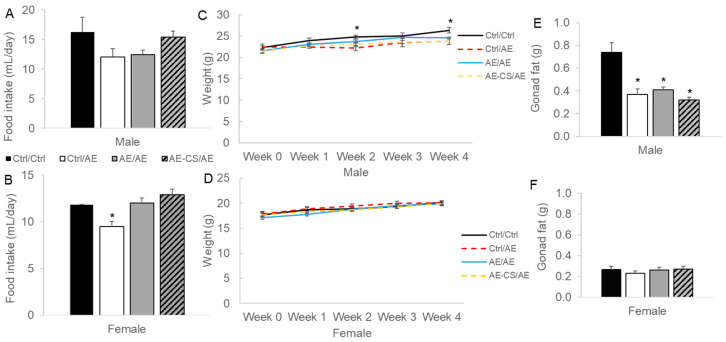
Food intake, weight gain, and gonadal fat weight of offspring mice exposed to ethanol with or without prenatal choline supplementation. C57BL/6J female mice received either a control or choline-supplemented diet with or without 1.4% ethanol during pregnancy. Their offspring (shown in this figure) received either a control or ethanol-containing (1.4% for females and 3.9% for males) Lieber–DeCarli liquid diet after weaning for 4 weeks. (**A**,**B**) average food intake of offspring during the 4-week post-weaning liquid diet feeding; (**C**,**D**) weight gain of offspring during the 4-week post-weaning liquid diet feeding; and (**E**,**F**) gonad fat weight of offspring after the 4-week post-weaning liquid diet feeding. Analyzed with one-way ANOVA. * *p* < 0.05. *n* = 6–8 offspring/sex/treatment group. AE: alcohol exposure; CS: choline supplementation; Ctrl: control. Ctrl/Ctrl: absolute control without alcohol exposure; Ctrl/AE: prenatal control and postnatal alcohol exposure; AE/AE: prenatal and postnatal alcohol exposure; AE-CS/AE: prenatal and postnatal alcohol exposure with prenatal choline supplementation.

Average food intake during the 4-week post-weaning control and ethanol liquid diet feeding was reduced in female offspring exposed to ethanol after weaning (Ctrl/AE) compared to other groups (*p* = 0.003), yet male offspring demonstrated no difference in the intake of these isocaloric diets (Figure 2).

We next compared the weight gain of the offspring after weaning and found that the postnatal AE male offspring had lower (*p* = 0.025) weight gain after the 4-week post-weaning control and ethanol liquid diet feeding than the unexposed absolute control (Ctrl/Ctrl) while there were no differences in the weight gain of female offspring (Figure 1). Gonad fat weight was reduced in all AE groups (*p* < 0.001) versus the Ctrl/Ctrl group in male but not female offspring (Figure 1). Prenatal AE did not have an independent effect on weight or gonad fat accumulation among the offspring.

### 3.2. Glucose Tolerance Was Improved in Prenatal Choline-Supplemented AE Male Offspring

**Figure 3:**
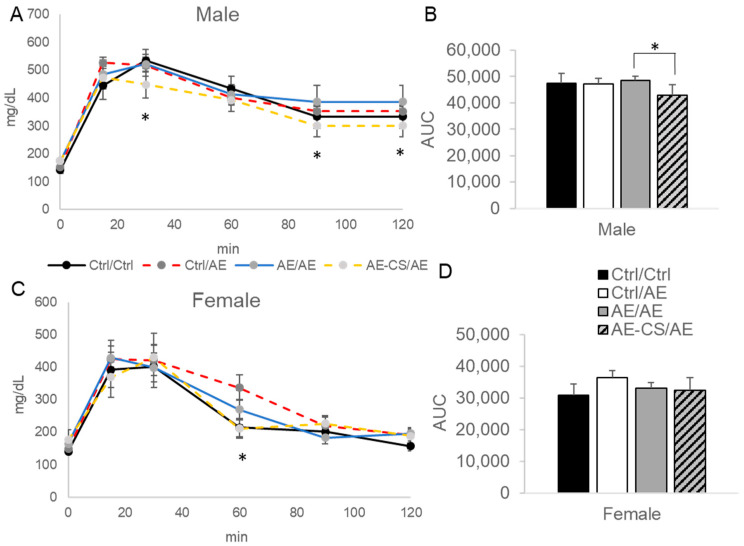
Glucose tolerance of offspring mice exposed to ethanol with or without prenatal choline supplementation. C57BL/6J female mice received either a control or choline-supplemented diet with or without 1.4% ethanol during pregnancy. Their offspring (shown in this figure) received either a control or ethanol-containing (1.4% for females and 3.9% for males) Lieber–DeCarli liquid diet after weaning for 4 weeks. Intraperitoneal glucose tolerance test (GTT) was conducted by intraperitoneal injection of 2 mg/g body weight of glucose and blood glucose monitoring in the following 2 h. (**A**,**B**) male offspring GTT curve and area under the curve (AUC) after 4-week post-weaning liquid diet feeding; (**C**,**D**) female offspring GTT curve and area under the curve after 4-week post-weaning liquid diet feeding. Analyzed with repeated measures ANOVA. * *p* < 0.05 AE-CS/AE compared to AE/AE for males and AE-CS/AE compared to Ctrl/AE for females. *n* = 6–8 offspring/sex/treatment group. AE: alcohol exposure; CS: choline supplementation; Ctrl: control. Ctrl/Ctrl: absolute control without alcohol exposure; Ctrl/AE: prenatal control and postnatal alcohol exposure; AE/AE: prenatal and postnatal alcohol exposure; AE-CS/AE: prenatal and postnatal alcohol exposure with prenatal choline supplementation.

We next examined glucose tolerance of the offspring and found that prenatal CS male offspring had better glucose tolerance than those without supplementation when they were both exposed to ethanol after weaning (AE-CS/AE vs. AE/AE, *p* < 0.05) (Figure 3). Female offspring did not show such a difference except for the 60 min post-injection time point, where the AE/AE group had higher blood glucose than the Ctrl/Ctrl group, which was prevented by CS in the AE-CS/AE group.

### 3.3. Liver TG Accumulation Was Exacerbated by AE and Prevented by Prenatal CS

Liver weights were not significantly different among the groups (Table 1). Hepatic histology also demonstrated no differences in the NAS among the groups. We then measured TG accumulation in the liver and found it to be elevated in the AE/AE male offspring than Ctrl/Ctrl which was normalized by prenatal CS in the AE-CS/AE group (*p* < 0.05). In female offspring, however, the Ctrl/AE offspring had elevated TG levels compared to Ctrl/Ctrl offspring, which was again normalized in the AE-CS/AE group (*p* < 0.05) (Table 1). Markers of oxidative stress measured by MDA levels in hepatic homogenate and liver damage assessed by serum ALT levels were not different among the groups in both male and female offspring (Table 1).

### 3.4. Liver Lipid Metabolic Gene Expression

**Figure 4:**
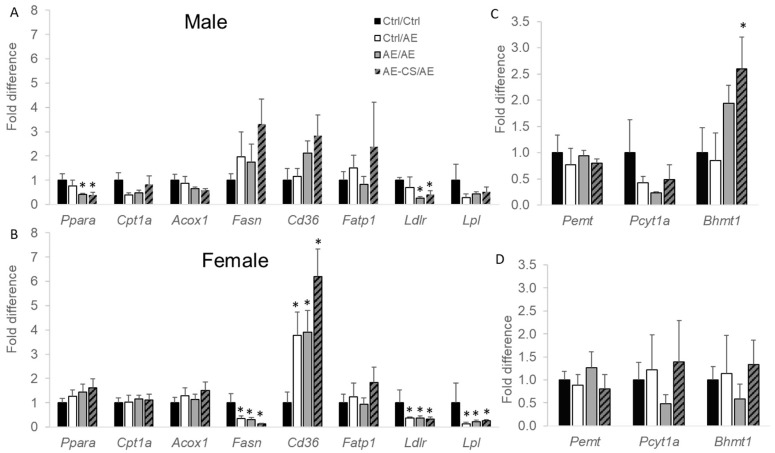
Liver gene expression of offspring mice exposed to ethanol with or without prenatal choline supplementation. C57BL/6J female mice received either a control or choline-supplemented diet with or without 1.4% ethanol during pregnancy. Their offspring (shown in this figure) received either a control or ethanol-containing (1.4% for females and 3.9% for males) Lieber–DeCarli liquid diet after weaning for 4 weeks. (**A**,**C**) male offspring liver gene expression after 4-week post-weaning liquid diet feeding; (**B**,**D**) female offspring liver gene expression after 4-week post-weaning liquid diet feeding. Analyzed with one-way ANOVA. * *p* < 0.05 compared to the Ctrl/Ctrl group. *n* = 6–8 offspring/sex/treatment group. AE: alcohol exposure; CS: choline supplementation; Ctrl: control. Ctrl/Ctrl: absolute control without alcohol exposure; Ctrl/AE: prenatal control and postnatal alcohol exposure; AE/AE: prenatal and postnatal alcohol exposure; AE-CS/AE: prenatal and postnatal alcohol exposure with prenatal choline supplementation. *Acox1*, Acyl-CoA oxidase 1; *Bhmt1*, betaine-homocysteine *S*-methyltransferase; *Cd36*, cluster of differentiation 36; *Cpt1a*, carnitine palmitoyltransferase 1a; *Fasn*, fatty acid synthase; *Fatp1*, fatty acid transporter 1; *Ldlr*, low-density lipoprotein receptor; *Lpl*, lipoprotein lipase; *Pcyt1a*, choline-phosphate cytidylyltransferase; *Pemt*, phosphatidylethanolamine *N*-methyltransferase; *Ppara*, peroxisome proliferator-activated receptor alpha.

To delineate the underlying mechanism by which excessive TG accumulation in the liver was prevented in prenatal CS offspring, we examined the mRNA expression of genes involved with lipid metabolism in the offspring liver. In male offspring, when they were exposed to ethanol both prenatally and postnatally (AE/AE), they demonstrated lower lipid catabolic gene *Ppara* and lipoprotein metabolic gene *Ldlr* expression compared to the Ctrl/Ctrl group (*p* < 0.05), and these differences were not observed between offspring that were only exposed to alcohol after weaning (Ctrl/AE) and the absolute control (Ctrl/Ctrl) (Figure 4). In female offspring, post-weaning AE groups had lower lipogenic gene *Fasn* expression, higher fatty acid transporter *Cd36* as well as lipoprotein metabolic gene *Lpl* and *Ldlr* expression than Ctrl/Ctrl (*p* < 0.05). Prenatal CS did not have significant effects on the expression of these genes (Figure 4).

### 3.5. TG, Free FFA, and ApoB Levels in Serum

Serum TG levels were unexpectedly higher in the AE-CS/AE group versus other groups in male offspring while there were no differences in female offspring (Table 1). FFA levels were similar among the groups (Table 1). We further measured ApoB levels, yet also did not find any significant differences among the groups (Table 1).

### 3.6. Hepatic Global DNA Methylation Was Not Altered by AE or CS

Since DNA methylation of the genome may be affected by AE, we measured global DNA methylation levels in the offspring liver, yet did not find any difference (Table 1).

### 3.7. Choline Metabolites and Gene Expression

In male offspring, exposure to ethanol during both the prenatal and postnatal periods increased phosphatidylcholine (PC) and lyso-PC levels while decreasing glycerophosphorylcholine (GPC) levels in the liver (*p* = 0.016 and *p* = 0.007, respectively) (Table 2). Prenatal CS increased hepatic-free choline levels (*p* = 0.019) but had no effects on the lipid-soluble choline derivatives. Postnatal AE in the Ctrl/AE group increased dimethylglycine levels in the liver compared to Ctrl/Ctrl, which was normalized by the combined effect of prenatal AE and CS (AE-CS/AE). In female offspring, GPC levels were increased in the AE-CS/AE group compared to Ctrl/Ctrl and AE/AE (*p* = 0.021 and *p* = 0.037, respectively). No other differences were observed.

We also explored the expression of choline metabolic genes. In male offspring, prenatal CS in the AE-CS/AE group increased the mRNA expression of *Bhmt1*, the enzyme that mediates the donation of the choline-derived methyl group for methylation reactions (*p* < 0.05) compared to the Ctrl/Ctrl and Ctrl/AE groups (Figure 4C). There were no differences in *Pcyt1a* or *Pemt* expression, the two enzymes that mediate the CDP-choline and de novo pathway of PC synthesis, respectively. There were also no differences in gene expression among the groups in female offspring (Figure 4D).

## 4. Discussion

In this study, we found that prenatal plus postnatal AE led to excessive TG accumulation in the liver of male mice. This could be normalized by prenatal CS. Prenatal CS also improved glucose tolerance in males. However, prenatal CS led to higher TG concentrations in circulation possibly due in part to the increased export of TG from the liver to circulation.

This is the first study that demonstrates that prenatal plus postnatal AE can result in excessive liver TG accumulation in male mice, which may increase the risk of fatty liver diseases. This observation is consistent with a two-hit theory of fatty liver diseases where prenatal AE exerts damage on the developing fetal liver, which increases its vulnerability to later AE that further damages hepatic regulation of lipid metabolism [37]. Gene expression analysis indicated that prenatal plus postnatal AE downregulated *Ppara* and *Ldlr*. PPAR-α is a critical nuclear factor and transcription modulator of lipid metabolism in the liver. It forms heterodimers with retinoid X receptors and binds to DNA sequences called PPAR response elements, and thereby controls the transcription of a series of genes such as CD36, ACOX1, and CPT1A that mediate fatty acid catabolism via β-oxidation [38,39]. It enhances fatty acid uptake into the liver for oxidation and alters lipoprotein-related gene expression to improve plasma lipid balance [38,39]. Deletion of *Ppara* results in steatosis during obesogenic feeding [40]. LDLR assists with the reuptake of cholesterol in the liver and prevents hypercholesterolemia [9,41]. Deletion of *Ldlr* is associated with morbid obesity in mice [42,43]. Our findings corroborated a previous study demonstrating reduced *Ldlr* expression and hypercholesterolemia in prenatal AE animals exposed to a postnatal HF diet, suggesting dysregulation of cholesterol metabolism resulting from the impaired reuptake of cholesterol in the liver in response to HF feeding in prenatal AE animals [9]. We also found that prenatal and postnatal AE increased PC concentrations in the liver, which could be a response to the increased demand for TG export via VLDL, which uses PC as a structural molecule. Our study further suggests that prenatal AE also renders additional susceptibility to cholesterol metabolism dysregulation under the condition of postnatal AE.

Our study also found that prenatal CS could normalize TG accumulation that was exacerbated by prenatal and postnatal AE. In addition, prenatal CS significantly improved glucose tolerance compared to AE/AE male offspring. These beneficial effects of prenatal CS were consistent with our previous observation in a mouse model of prenatal and postnatal obesogenic feeding. In those studies, female mice were fed an HF diet throughout gestation and their offspring were also exposed to HF feeding after weaning. We found that maternal CS not only prevented placental and fetal overgrowth at embryonic day (E) 12.5 due to HF feeding [32], but also normalized fetal adiposity and liver TG content at E17.5 [30]. After 6-week HF feeding post-weaning, the male offspring also had improved glucose tolerance than their unsupplemented counterparts [31]. How maternal CS improved metabolic functioning may be attributed to its roles in lipid metabolism. At E17.5, maternal CS suppressed the expression of several de novo lipogenic genes such as *Srebp1c* in the fetal liver [30]. These changes could be related to the function of choline as a methyl donor since both fetal liver global DNA methylation and promoter methylation of *Srebp1* were increased by maternal CS [44]. However, in the current study, we did not find a clear molecular mechanism by which maternal CS normalized hepatic TG content since neither of the genes related to lipogenesis nor catabolism were altered. Alternatively, CS may have facilitated hepatic TG export to circulation through its role as a structural component of lipoproteins. This hypothesis was supported by the increased TG in circulation in the AE-CS/AE group. However, neither of the measurements of ApoB, a marker of VLDL and LDL, in circulation nor gene expression related to lipoprotein metabolism (e.g., *Lpl* and *Ldlr*) demonstrated differences by prenatal CS. Further research is also needed to delineate the long-term influence of prenatal CS on overall metabolic health, with this reduced lipid load in the liver yet potentially heightened burden in extrahepatic tissues.

Epigenetic modification is a central mechanism by which the intrauterine environment leaves a lasting mark on the offspring that is maintained into postnatal life [45]. AE reduces DNA methylation by depleting the universal methyl donor SAM in the liver in a previous report [21]. Methyl donor supplementation of betaine or folate rectifies the decrease in SAM and deregulated lipid metabolism, respectively [21,46]. Our study, however, did not find an effect of prenatal CS on global DNA methylation of AE offspring livers, unlike our previous report on the mouse model with prenatal CS and HF feeding [44]. It is, however, still possible that some susceptible loci such as those in the growth pathway (e.g., insulin-like growth factor 2) might be affected by prenatal CS during AE, which requires further exploration.

This study also demonstrated that both prenatal and postnatal AE altered liver choline metabolite levels. Specifically, postnatal AE increased dimethylglycine levels, which indicated increased use of betaine (the oxidized product of choline) as a methyl donor. Interestingly, prenatal CS in the context of AE enhanced the expression of *Bhmt1*, which mediates the reaction of methyl group donation from betaine to homocysteine forming dimethylglycine and methionine, after 4 weeks of postnatal AE. However, liver dimethylglycine levels in this prenatal CS group were normalized to those of the unexposed absolute control Ctrl/Ctrl. Overall, it seems to suggest that postnatal AE dysregulated the participation of choline in one-carbon metabolism, which was normalized by prenatal CS via the upregulation of BHMT. In addition, the combined effect of prenatal and postnatal AE resulted in elevated PC and lyso-PC, in addition to decreases in GPC, a metabolic profile favoring PC synthesis. The increase in PC may be a mechanism of adjustment of the body to AE by increasing hepatic TG export via PC-containing VLDL. Nevertheless, we did not find any differences in the expression of rate-limiting enzymes *Pcyt1a* and *Pemt* in the CDP-choline and de novo PC synthesis pathways. Compared to a previous study that our group conducted [44], the above alterations were different from those that were induced by prenatal and postnatal HF feeding, despite the fact that both AE and HF result in fatty liver diseases. We found in that study that postnatal HF feeding depleted methionine and PC, while prenatal HF had no additional effects.

There are substantial differences in response to prenatal CS and AE between males and females, consistent with other studies of AE and prenatal programming [9]. However, it should be noted that because female mice had a lower tolerance to AE as demonstrated by the rapid weight loss with AE higher than 1.4% ethanol in food, we fed female offspring with 1.4% ethanol while male offspring were on a 3.9% ethanol diet. These different dosages of exposure may provide an alternative explanation to the differential responses we observed. Specifically, AE led to lower weight gain in male but not female offspring. Prenatal AE led to significantly higher TG contents in male but not female offspring. In addition, the male offspring demonstrated altered *Ppara* and *Ldlr* expression with the combined effect of prenatal and postnatal AE, while in female offspring, postnatal AE alone was sufficient to downregulate *Ldlr* expression. Overall, it seems that male offspring or those with a higher AE dosage were more susceptible to prenatal AE and responsive to CS [31,44,47]. In a prior study of 4 g/kg prenatal AE only, female offspring were found to have a greater reduction in SR-B1 and CYP7A1 cholesterol metabolic gene expression and thus potentially more susceptible to cholesterol dysregulation [9]. Further studies that pay attention to sexually dimorphic response, dosage, and timing of AE are important for both the advancement of understanding of this topic and drug development.

This study has some limitations. The need to use different dosages for ethanol for the two sexes may have confounded the examination of sexually dimorphic responses to ethanol. The prepregnancy and gestational choline supplementation dosages were slightly different at 4.5 times and 4 times the control, respectively. Nevertheless, a maternal choline intake that was 4 times higher than the control was sufficient to improve various outcomes of rodent offspring in prior studies [48] and thus the minor difference between the two dosages was not likely to alter our metabolic outcomes of interest. It was a short-term postnatal study of ethanol exposure that did not follow for a long enough time period until full-blown alcoholic liver disease develops. In addition to the liquid diets, an additional single binge ethanol exposure of 5 g/kg via gavage feeding may accelerate the development of hepatic inflammation [33]. There was also a need to include a postnatal CS group to determine the appropriate timing of CS for those with prenatal and postnatal AE.

## 5. Conclusions

In conclusion, prenatal plus postnatal AE seems to increase the metabolic damage in offspring males, which can be partially normalized by prenatal CS. Further studies are needed to pinpoint the mechanisms and delineate the influence of timing of exposure, dosage of treatment, and tissue and sex-specific patterns.

## Figures and Tables

**Table 1:** Liver and serum biomarkers of offspring in the different dietary treatment groups.

	Ctrl/Ctrl	Ctrl/AE	AE/AE	AE-CS/AE
	Male			
Number of animals	*n* = 6	*n* = 8	*n* = 7	*n* = 7
Liver weight (g)	1.13 ± 0.15	1.14 ± 0.18	1.20 ± 0.11	1.25 ± 0.22
Liver NAS	0.58 ± 0.39	1.69 ± 0.94	1.28 ± 0.33	2.67 ± 0.92
Liver triglyceride (mg/g)	11.5 ± 2.5 ^a^	16.9 ± 2.8 ^a,b^	22.6 ± 4.4 ^b^	13.9 ± 1.4 ^a^
Liver MDA (nM/g)	51.2 ± 15.0	44.0 ± 7.9	59.8 ± 10.1	55.5 ± 12.5
Serum ALT (IU/L)	37.3 ± 13.3	72.6 ± 24.4	34.8 ± 10.5	105.6 ± 17.0
Serum triglyceride (mg/dL)	45.4 ± 10.5 ^b^	69.0 ± 10.9 ^b^	60.3 ± 12.1 ^b^	107.3 ± 12.6 ^a^
Serum FFA (nmol/L)	0.62 ± 0.09	0.86 ± 0.22	0.69 ± 0.10	0.89 ± 0.10
Serum ApoB (µg/mL)	26.2 ± 16.6	11.6 ± 5.2	12.4 ± 2.2	19.6 ± 8.6
Global DNA methylation (fold difference)	1.00 ± 0.68	0.54 ± 0.18	0.27 ± 0.09	0.80 ± 0.31
	Female			
Number of animals	*n* = 6	*n* = 8	*n* = 6	*n* = 6
Liver weight (g)	0.99 ± 0.04	0.94 ± 0.04	0.95 ± 0.03	0.88 ± 0.04
Liver NAS	0.25 ± 0.25	0.50 ± 0.19	0.23 ± 0.14	0.58 ± 0.31
Liver triglyceride (mg/g)	8.3 ± 1.4 ^a^	19.4 ± 2.7 ^b^	13.3 ± 2.0 ^a,b^	13.2 ± 1.6 ^a^
Liver MDA (nM/g)	58.5 ± 8.7	40.0 ± 5.3	40.6 ± 3.8	80.8 ± 33.5
Serum ALT (IU/L)	32.5 ± 9.5	64.0 ± 43.2	17.2 ± 3.3	41.8 ± 11.2
Serum triglyceride (mg/dL)	53.7 ± 5.3	63.9 ± 21.0	55.6 ± 5.5	45.8 ± 7.3
Serum FFA (nmol/L)	0.66 ± 0.10	0.91 ± 0.46	0.85 ± 0.30	0.87 ± 0.36
Serum ApoB (µg/mL)	6.6 ± 4.3	6.5 ± 3.3	10.2 ± 2.5	10.4 ± 1.5
Global DNA methylation (fold difference)	1.00 ± 0.22	0.62 ± 0.45	0.49 ± 0.19	0.56 ± 0.12

Analyzed with one-way ANOVA; C57BL/6J female mice received either a control or choline-supplemented diet with or without 1.4% ethanol during pregnancy. Their offspring (shown in this table) received either a control or ethanol-containing (1.4% for females and 3.9% for males) Lieber–DeCarli liquid diet after weaning for 4 weeks. ^a,b^ different letters indicate significant difference, *p* < 0.05. *n* = 6–8 offspring/sex/treatment group. AE: alcohol exposure; CS: choline supplementation; Ctrl: control. Ctrl/Ctrl: absolute control without alcohol exposure; Ctrl/AE: prenatal control and postnatal alcohol exposure; AE/AE: prenatal and postnatal alcohol exposure; AE-CS/AE: prenatal and postnatal alcohol exposure with prenatal choline supplementation. ALT: alanine transaminase; ApoB: apolipoprotein B; FFA: free fatty acid; MDA: malondialdehyde; NAS: nonalcoholic fatty liver disease activity score.

**Table 2:** Offspring liver choline metabolites in the different dietary treatment groups.

	Ctrl/Ctrl	Ctrl/AE	AE/AE	AE-CS/AE
	Male (nmol/g)			
Number of animals	*n* = 6	*n* = 8	*n* = 7	*n* = 7
Methionine	191 ± 25	278 ± 46	228 ± 23	265 ± 21
Choline	387 ± 116 ^a^	301 ± 68 ^a^	606 ± 24 ^a,b^	869 ± 184 ^b^
Dimethylglycine	19.6 ± 0.8 ^a^	48.8 ± 10.6 ^b^	33.5 ± 8.8 ^a,b^	26.9 ± 4.3 ^a^
Betaine	205 ± 51	581 ± 302	240 ± 63	182 ± 48
Trimethylamine-oxide	1.3 ± 0.2 ^b^	0.8 ± 0.3 ^a,b^	0.7 ± 0.2 ^a,b^	0.4 ± 0.3 ^a^
Glycerophosphorylcholine	711 ± 204 ^b^	429 ± 91 ^a,b^	285 ± 37 ^a^	227 ± 65 ^a^
Phosphorylcholine	81 ± 23	105 ± 16	144 ± 23	145 ± 28
Phosphatidylcholine	16,495 ± 450 ^a^	20,343 ± 2728 ^a,b^	23,575 ± 716 ^b^	23,527 ± 789 ^b^
Sphingomyelin	1727 ± 80 ^a^	2566 ± 226 ^c^	1910 ± 90 ^a,b^	2282 ± 190 ^b,c^
Lysophosphatidylcholine	562 ± 23 ^a^	598 ± 63 ^a^	728 ± 14 ^b^	754 ± 28 ^b^
	Female (nmol/g)			
Number of animals	*n* = 6	*n* = 8	*n* = 6	*n* = 6
Methionine	152 ± 13	182 ± 17	223 ± 24	164 ± 37
Choline	214 ± 58	212 ± 55	289 ± 124	194 ± 56
Dimethylglycine	39.7 ± 7.5	40.4 ± 10.4	32.9 ± 4.0	34.3 ± 1.6
Betaine	354 ± 103	467 ± 96	399 ± 58	531 ± 59
Trimethylamine-oxide	38.4 ± 17.9	14.1 ± 2.3	19.0 ± 5.0	13.2 ± 4.5
Glycerophosphorylcholine	372 ± 26 ^a^	490 ± 74 ^a,b^	412 ± 60 ^a^	721 ± 157 ^b^
Phosphorylcholine	80 ± 30	113 ± 32	115 ± 24	207 ± 73
Phosphatidylcholine	20,180 ± 1037	20,503 ± 823	19,698 ± 1138	18,393 ± 1296
Sphingomyelin	1990 ± 213	1998 ± 104	1875 ± 84	1792 ± 179
Lysophosphatidylcholine	646 ± 54	629 ± 24	654 ± 19	577 ± 44

C57BL/6J female mice received either a control or choline-supplemented diet with or without 1.4% ethanol during pregnancy. Their offspring (shown in this table) received either a control or ethanol-containing (1.4% for females and 3.9% for males) Lieber–DeCarli liquid diet after weaning for 4 weeks. Analyzed with one-way ANOVA. ^a,b,c^ different letters indicate significant difference, *p* < 0.05. *n* = 6–8 offspring/sex/treatment group. AE: alcohol exposure; CS: choline supplementation; Ctrl: control. Ctrl/Ctrl: absolute control without alcohol exposure; Ctrl/AE: prenatal control and postnatal alcohol exposure; AE/AE: prenatal and postnatal alcohol exposure; AE-CS/AE: prenatal and postnatal alcohol exposure with prenatal choline supplementation.

## Data Availability

Data will be available upon request.

## Supplementary Materials

The following supporting information can be downloaded at: https://www.mdpi.com/article/10.3390/nu16091264/s1. Supplementary Table S1. Dietary composition of liquid diets.
