# Supplementary material for: Prenatal Choline Supplementation Improves Glucose Tolerance and Reduces Liver Fat Accumulation in Mouse Offspring Exposed to Ethanol during the Prenatal and Postnatal Periods
Source: Nutrients. 2024 Apr 24;16(9):1264. doi: 10.3390/nu16091264 (PMC11085373; doi:10.3390/nu16091264)
Supplement: Supplementary file 1 [file nutrients-16-01264-s001.zip › nutrients-2958038-supplementary.pdf]

Supplementary Table S1. Composition of Lieber-DeCarli’82 liquid diets used in the study

	Liquid control	1.4% ethanol	3.9% ethanol	1.4% ethanol + choline
Protein (kcal/L)	151	151	151	151
Fat (kcal/L)	359	359	359	359
Carbohydrate (kcal/L)	490	135	135	135
Ethanol (mL, kcal/L)	0	17.8, 99.4	49.7, 276.9	49.7, 276.9
Maltose dextrin (g, kcal/L)	0	65, 255.6	20, 78.1	20, 78.1
Choline (mg/L)	206	206	206	825
Total calorie (kcal/L)	1000	1000	1000	1000

Lieber-DeCarli’82 control (F1259SP) and Lieber-DeCarli’82 ethanol (F1258SP) diets were purchased from Bio-serv, Flemington, NJ, USA. Diets were received in the form of powder and reconstituted in water before use. Ethanol, maltose dextrin, and additional choline were added during preparation of the liquid diets.
